# Promise from the Sea

**DOI:** 10.3390/md14100178

**Published:** 2016-10-09

**Authors:** George Perry

**Affiliations:** College of Sciences, University of Texas at San Antonio, San Antonio, TX 79249-0661, USA; George.Perry@utsa.edu; Tel.: +1-210-458-4450

The twenty-first century’s greatest medical challenge is degenerative disease. In the twentieth century, effective treatment and cures were developed for infections, diseases of defect, and injuries; however, our efforts with age-related degeneration have, at most, been to promote stability. Nowhere is this clearer than with neurodegenerative diseases; the current therapies improve quality of life by only a small measure. The field is calling for drugs of novel modes of action. The greatest promise is likely to come from drugs targeted to physiological pathways—natural products. A prime contender is the sea, where animal and plant diversity is greatest and least explored. In this Issue, we present leading edge articles showing the great promise of the oceans, and the need to further explore this promise.

